# Prognostic mutation signature would serve as a potential prognostic predictor in patients with diffuse large B-cell lymphoma

**DOI:** 10.1038/s41598-024-56583-4

**Published:** 2024-03-14

**Authors:** Shih-Feng Cho, Tsung-Jang Yeh, Hui-Ching Wang, Jeng-Shiun Du, Yuh-Ching Gau, Yu-Yin Lin, Tzer-Ming Chuang, Yi-Chang Liu, Hui-Hua Hsiao, Sin-Hua Moi

**Affiliations:** 1 Division of Hematology & Oncology, Department of Internal Medicine, Kaohsiung Medical University Hospital, Kaohsiung Medical University, Kaohsiung, 807 Taiwan; 2 Faculty of Medicine, College of Medicine, Kaohsiung Medical University, Kaohsiung, 807 Taiwan; 3 Center for Cancer Research, Kaohsiung Medical University, Kaohsiung, 807 Taiwan; 4 Center for Liquid Biopsy and Cohort Research, Kaohsiung Medical University, Kaohsiung, 807 Taiwan; 5 Graduate Institute of Clinical Medicine, College of Medicine, Kaohsiung Medical University, Kaohsiung, 807 Taiwan; 6 Health Management Center, Kaohsiung Medical University Hospital, Kaohsiung Medical University, Kaohsiung, 807 Taiwan; 7 Research Center for Precision Environmental Medicine, Kaohsiung Medical University, Kaohsiung, 807 Taiwan; 8 Department of Medical Research, Kaohsiung Medical University Hospital, Kaohsiung Medical University, Kaohsiung, 807 Taiwan

**Keywords:** Diffuse large B-cell lymphoma, Somatic mutation, Prognostic mutation signature, Non-hodgkin lymphoma, Cancer genomics, Prognostic markers

## Abstract

The present study aimed to elucidate the prognostic mutation signature (PMS) associated with long-term survival in a diffuse large B-cell lymphoma (DLBCL) cohort. All data including derivation and validation cohorts were retrospectively retrieved from The Cancer Genome Atlas (TCGA) database and whole-exome sequencing (WES) data. The Lasso Cox regression analysis was used to construct the PMS based on WES data, and the PMS was determined using the area under the receiver operating curve (AUC). The predictive performance of eligible PMS was analyzed by time-dependent receiver operating curve (ROC) analyses. After the initial evaluation, a PMS composed of 94 PFS-related genes was constructed. Notably, this constructed PMS accurately predicted the 12-, 36-, and 60-month PFS, with AUC values of 0.982, 0.983, and 0.987, respectively. A higher level of PMS was closely linked to a significantly worse PFS, regardless of the molecular subtype. Further evaluation by forest plot revealed incorporation of international prognostic index or tumor mutational burden into PMS increased the prediction capability for PFS. The drug-gene interaction and pathway exploration revealed the PFS-related genes were associated with DNA damage, TP53, apoptosis, and immune cell functions. In conclusion, this study utilizing a high throughput genetic approach demonstrated that the PMS could serve as a prognostic predictor in DLBCL patients. Furthermore, the identification of the key signaling pathways for disease progression also provides information for further investigation to gain more insight into novel drug-resistant mechanisms.

## Introduction

Diffuse large B-cell lymphoma (DLBCL) is the most common aggressive non-Hodgkin lymphoma (NHL) worldwide, accounting for approximately 30–40% of annual newly diagnosed lymphoma cases^1,2^. The incorporation of the anti-CD20 monoclonal antibody, rituximab (R), into conventional anthracycline-based chemotherapy results in a high response rate and prolonged overall survival of patients with DLBCL^3–5^. For patients with newly diagnosed DLBCL, the standard R-CHOP (cyclophosphamide, doxorubicin, vincristine, and prednisone) regimen achieves a high complete remission rate (approximately 75%), and 60–70% of these patients remain relapse-free after 5 years of follow-up^6,7^. However, a proportion of DLBCL patients experience primary refractory disease or relapse after prior successful treatment. The prognosis of this subgroup is dismal, making the exploration of resistance mechanisms or new therapies an urgent medical need.

Accumulating evidence suggests that highly heterogeneous genetic alterations and the tumor microenvironment play crucial roles in treatment failure^8^. The gene expression profiling divides DLBCL into two distinct groups, namely, germinal center B-cell-like (GCB) and activated B-cell-like (ABC). Patients with ABC-DLBCL tend to have a poorer prognosis and a higher risk of treatment resistance^9–11^. In addition, a small subset of patients with MYC, BCL2, and/or BCL6 arrangement has been classified as double-hit lymphoma (DHL) or triple-hit lymphoma (THL), these patients with DHL/THL tend to respond poorly to R-CHOP regimen^12–14^. Advances in next-generation sequencing (NGS) technology and bioinformatics allow integrative genomic analyses in a large cohort of patients, enabling the identification of novel genetic subsets and modeling of novel genetic classifications^15–17^. Several genetic alterations related to relapsed or refractory DLBCL after R-CHOP treatment were identified, including epigenetic regulation, cell cycle regulation, signaling pathway activation, and oncogenes^18–21^. Moreover, the germinal center-related microenvironmental signature stratified DLBCL patients into different risk groups after R-CHOP treatment^22^.

Several models or genetic soring systems incorporating integrative gene expression analyses are under development, aiming to provide better prognostic information^23^. A four-gene signature-based score involving immune infiltration separated patients into high- and low-risk groups. Notably, the combination of the gene expression-based score with the international prognostic index (IPI) further improved the risk prediction^24^. Another study investigating NGS data has shown that MYC/BCL2, microenvironment biomarkers, and genetic subtyping are closely linked to the clinical outcome of DLBCL patients^25^. Despite these findings, investigation of gene mutation patterns and the degree of gene expression in the TME to explore new prognostic markers and novel therapeutic targets are still very critical.

In the present study, whole-exome sequencing (WES) data from a cohort of DLBCL patients was investigated and further validated by the TCGA database. In addition to the estimated tumor mutational burden, a panel composed of several prespecified gene expression signatures was incorporated as a panel of functional genes. These genes were then harnessed to formulate somatic mutation profiles that hold relevance to the prognosis of the disease, henceforth referred to as the prognostic mutation signature (PMS). Overall, this study aims to assess the utilization of both mutational signatures and common clinicopathological characteristics on prognostic outcomes for DLBCL patients.

## Methods

### Data source

**Figure 1:**
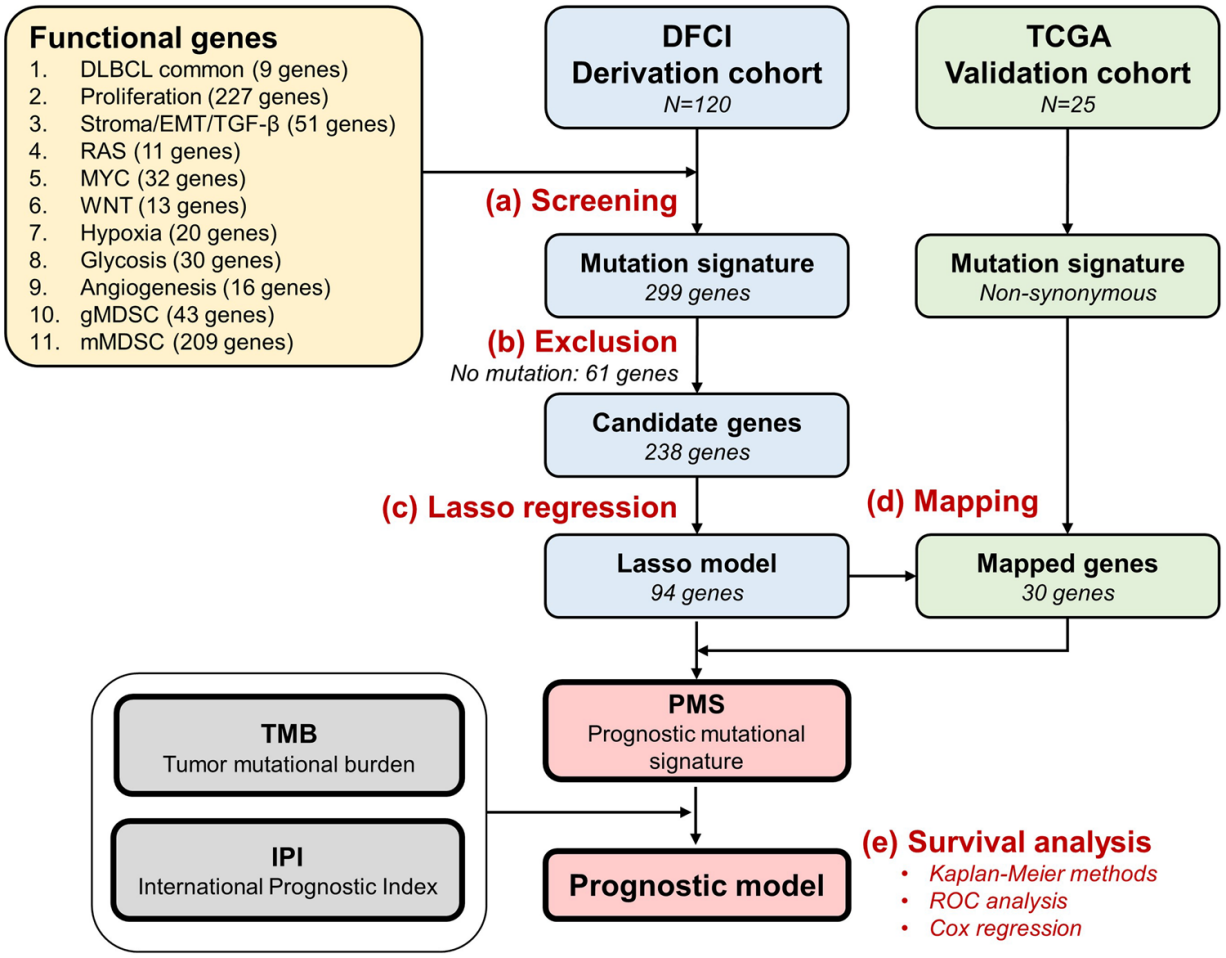
The study flowchart. (a) The genes in the eleven gene sets were screened by utilizing the data from whole exome sequencing in the DFCI cohort. (b) A total of 299 genes were identified for the mutation signature evaluation. Then sixty-one genes without mutations were excluded, resulting in 238 candidate genes. (c) Using Lasso regression, a total of 94 genes related to long-term survival were identified. (d) For validation, another cohort (TCGA) was utilized, and thirty genes were mapped. (e) Finally, the prognostic mutational signature (PMS) was constructed for further investigation.

The DFCI dataset of DLBCL patients (DFCI, Nat Med 2018)^16^ encompassing clinicopathological characteristics and genomic data was collected for the derivation dataset, which was accessed through cBioPortal (http://www.cbioportal.org). The DFCI derivation cohort consisted of 135 patients with DLBCL including 120 patients who underwent standard R-CHOP therapy. The clinicopathological characteristics, somatic mutation profiles, and survival outcomes of the study cohort were also acquired. Furthermore, the data of DLBCL patients from TCGA database was also collected as the validation cohort. The TCGA validation cohort consisted of 48 patients with DLBCL including 25 patients who ever treated with CHOP-based therapy. The study flowchart is presented in Fig. 1.

The clinicopathological characteristics included age at diagnosis, sex, molecular subtype, and IPI (international prognostic index) score. Progression-free survival (PFS) was defined as the time from first treatment until disease progression or death. Because most of the relapses of DLBCL were observed within the first 2 years of diagnosis and the progression rate at five years was low, we observed the five-year survival outcome of the study cohort^26,27^. Patients who experienced relapse/progression of the disease or died within the study observed period (5 years) were considered the “Cases” group, and patients who achieved durable progression-free status were considered the “Controls” group.

### Somatic mutation profiles

The somatic mutation profiles were obtained from the WES of tumor-normal matching sample pairs, and the details of the samples and WES procedure have been well described in a previous publication^16^. The number of genetic mutations in the cancer cells was computed and is presented as tumor mutational burden (TMB, mut/MB). The somatically mutated status of each gene in candidate functional gene sets was selected and used to derive a PMS for the study cohort according to their PFS status.

### Functional gene sets

To investigate the complex genetic event and tumor microenvironment, a panel containing 11 gene functional signatures was utilized^28^, including DLBCL common genes, proliferation, stroma/EMT/TGF-β, RAS, MYC, WNT, hypoxia, glycolysis, angiogenesis, gMDSC (granulocytic myeloid-derived suppressor cells), and mMDSC (monocytic myeloid-derived suppressor cells) gene sets. The frequent mutated genes in DLBCL, including BCL2, TP53, MYC, MCM5, TSHZ3, KLHL6, MYD88, CD79B, and CREBBP, were defined as DLBCL common gene set (Supplementary Table S1).

### Prognostic mutational signature (PMS)

The Lasso Cox model is particularly suitable for high-dimensional somatic mutation data due to its ability to handle sparsity, prevent overfitting, and provide a more interpretable and relevant subset of genomic features for predicting survival outcomes in the context of cancer genomics. Lasso Cox regression analysis was performed to select the optimal gene combination for prognostic risk prediction. The optimal gene combination of candidate functional genes was selected using the Lasso Cox model. The somatic mutation rate of the selected genes in study cohorts was summarized using oncoprints. Afterward, the estimated coefficients ($\beta$) of eligible genes in optimal gene combination were computed using Cox regression. The $\beta$ and mutation status (*mut*) of each gene (*g*) were used to generate the candidate prognostic mutational signature (*PMS*) according to Eq. (1) as follows:

1 $$PMS= \sum_{g=1}^{n}{\beta }_{g}{ \times mut}_{g} , { mut}_{g}= \left\{\begin{array}{c}0=wild\\ 1=mutated\end{array}.\right.$$

The time-dependent prognostic predictive performance at 12-, 36-, and 60-months of derived PMS, TMB, and IPI were evaluated using ROC analysis, and the area under the ROC curve (AUC) was reported. The threshold values in the AUC for ROC analysis represent the spectrum of sensitivity and specificity trade-offs. The AUC represents the overall discriminatory power of the corresponding feature, while AUC thresholds of 0.7, 0.8, and 0.9 indicate acceptable, good, and excellent dichotomous predictive performance. In addition, the PMS was dichotomized into corresponding low- and high-PMS subgroups according to the optimal cutoff point estimated by ROC. The survival difference between the low- and high-PMS subgroups was further evaluated using the Kaplan‒Meier estimator and tested using the log-rank test.

### Statistical analyses

The clinicopathological characteristics, somatic mutation profiles, and survival outcomes were summarized and the difference between Cases and Controls groups was estimated using chi-squared, Fisher’s exact test, or Wilcoxon rank-sum test. Univariate and multivariate Cox proportional hazard regression analyses were performed to evaluate the association between PFS and PMS, while TMB and IPI were considered as covariates for model adjustment. The estimated Cox models were further summarized and illustrated using the forest plot. All *p* values were two-sided, and *p* < 0.05 was considered statistically significant. All analyses were conducted using R 4.1.2^29^.

## Results

### Baseline characteristics of the study cohort

The clinicopathological characteristics, TMB, somatic mutation, and all-cause mortality status of the DFCI derivation cohort according to PFS status are summarized in Supplementary Table S2. There were 52 patients with disease progression (Cases group) and 68 progression-free patients (Controls group). The case group was older and had a higher proportion of females. Both groups had a similar percentage in the molecular subtype. The Cases group also showed a higher proportion of high IPI scores than the Controls group. Notably, 42 (80.8%) patients in the Cases group died during the follow-up period. The basic characteristics of the TCGA validation cohort were also summarized (Supplementary Table S3). The distribution of clinicopathological characteristics, TMB, and survival status between Cases and Controls from the TCGA validation cohort did not show a significant difference.

### Somatic mutation profiles

The somatic mutation of the derivation cohort (n = 120) was first investigated. The most common somatic mutations were BCL2, TP53, and CREBBP (Supplementary Table S1). Among the patients with the ABC subtype, the most frequent mutations are in MYD88, CD79B, and TP53. In the GCB group, the most frequent mutations included BCL2, TP53, and CREBBP. Besides, BCL2, CREBBP, and TP53 are the most mutated genes in the unclassified group (Supplementary Table S1).

Next, to investigate survival-related somatic mutations, the genes in the 11 gene sets were first selected, and then the selected genes without any mutation variants in this study cohort were excluded. After the initial evaluation, the candidate genes were selected for further analysis, including DLBCL common (9 genes), proliferation (75 genes), stroma/EMT/TGF-β (34 genes), RAS (2 genes), MYC (8 genes), WNT (9 genes), hypoxia (4 genes), glycolysis (8 genes), angiogenesis (3 genes), gMDSC (15 genes), and mMDSC (80 genes).

### Significant prognostic mutational signature

**Figure 2:**
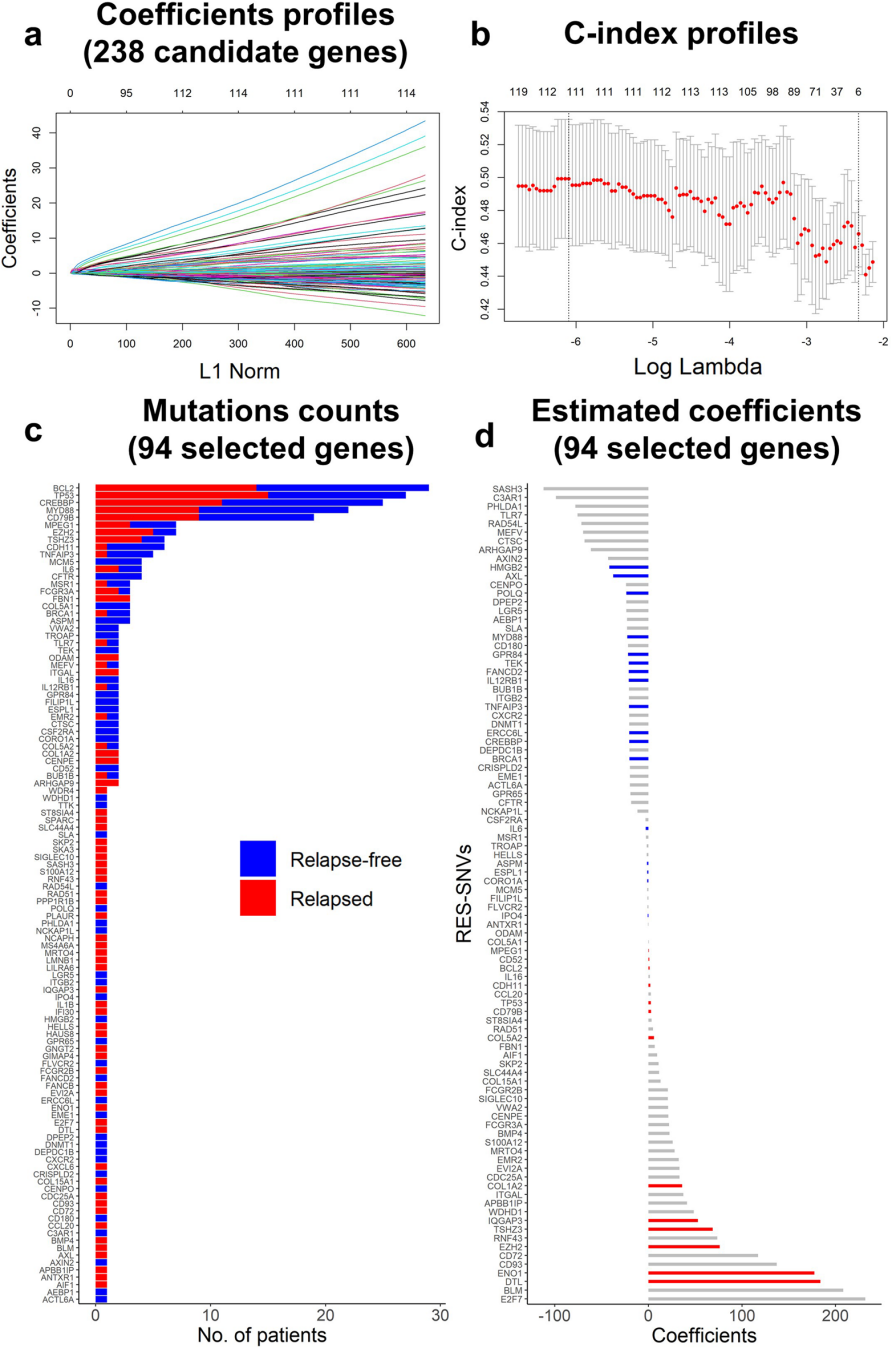
Lasso Cox regression analysis results. (**a**) Coefficients profiles of 238 candidate functional genes. (**b**) C-index profiles of estimated gene combinations. (**c**) The mutation rate of 94 selected genes according to relapse status, the blue bar indicates mutated relapse-free patients, and the red bar indicates mutated relapsed patients. (**d**) Estimated coefficients of 94 selected genes for PMS estimation (See details information in Table S4).

**Figure 3:**
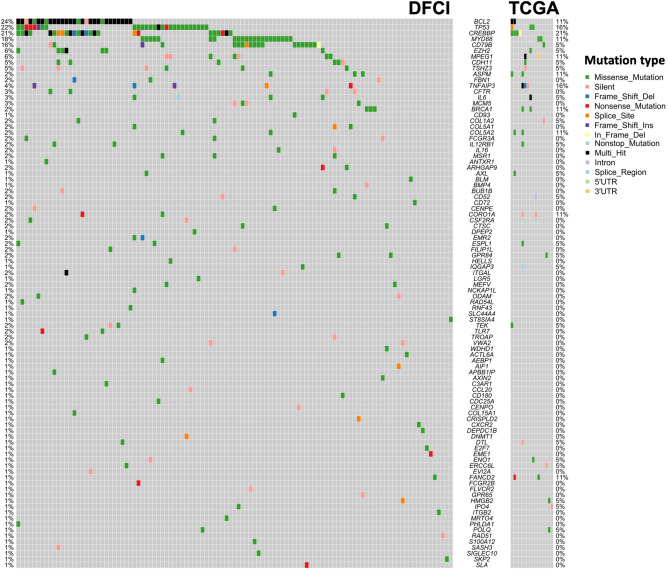
Oncoprint of 94 PMS genes in DFCI derivation cohort and TCGA validation cohort.

The results of the Lasso Cox regression analysis are shown in Fig. 2. The optimal gene combination for prognostic risk prediction was selected based on the log lambda (λ) validation obtained from the Lasso Cox model (Fig. 2a,b). We demonstrated that the estimated C-index from 94 genes combination exhibited optimal prediction performance (Fig. 2b). Figure 2c summarizes the mutation rate of 94 selected genes for PMS estimation in both relapse-free (blue bar) and relapse (red bar) patients, employing stacked bar plots. In the DFCI derivation cohort, *BCL2*, *TP53*, *CREBBP2*, *MYD88*, and *CD79B* were the five most common mutated genes (Fig. 2c). The $\beta$-value (standardized regression coefficients) of each gene in the optimal gene combination was presented in Fig. 2d, and the details were summarized in Supplementary Table S4. There were 51 genes related to an elevated risk of progression, whereas 43 genes showed a lower risk of progression (Fig. 2d). The somatic mutation profiles of the 94 PMS genes in both DFCI derivation and TCGA validation cohorts were also illustrated using oncoprints (Fig. 3). The common mutated genes in DFCI and TCGA cohorts include *BCL2* (DFCI: 24%, TCGA: 11%), TP53 (DFCI: 22%, TCGA: 16%), *CREBBP2* (DFCI: 21%, TCGA: 21%), *MYD88* (DFCI: 18%, TCGA: 11%), and *CD79B* (DFCI:16%, TCGA:5%).

### Predictive performance of the prognostic model

**Figure 4:**
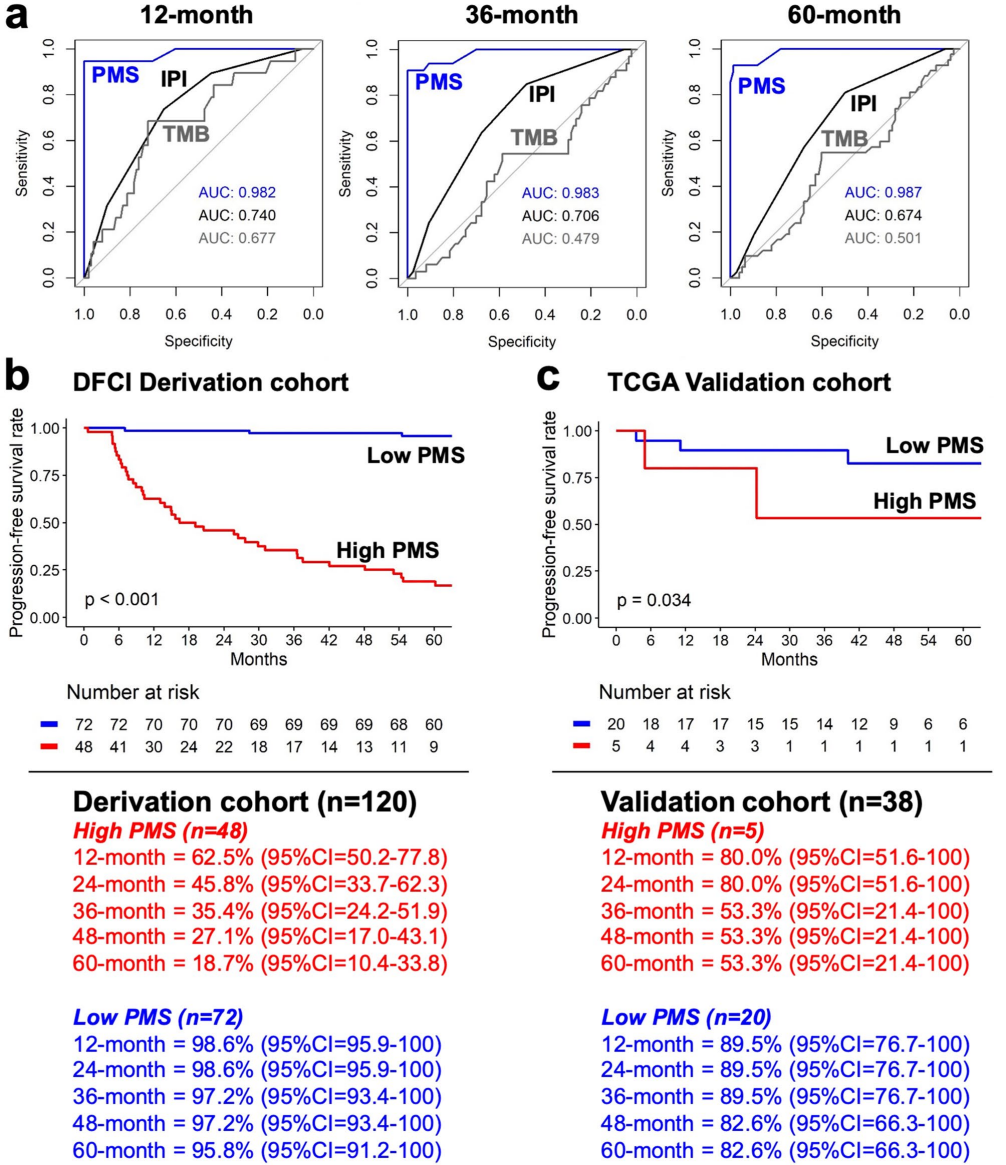
The predictive performance of PMS. (**a**) Time-dependent ROC curve using the PMS (blue), TMB (grey), and IPI (black) to predict 12-month, (B) 36-month, and (C) 60-month PFS. Kaplan‒Meier plot of low-and high-PMS in (**b**) DFCI derivation cohort and (**c**) TCGA validation cohort.

The results of the time-dependent ROC analysis for the prognostic prediction of PMS, TMB, and IPI at 12-, 36-, and 60-months PFS are shown in Fig. 4a. Notably, the time-dependent AUC values of PMS (AUCs: 0.982 to 0.987) increased over time, while the time-dependent AUC values of TMB (AUCs: 0.677 to 0.501) and IPI (AUCs: 0.740 to 0.674) decreased slightly. Moreover, PMS also obtained better predictive ability for both short-term and long-term PFS prediction compared to TMB and IPI. Based on AUC evaluation, the optimal cutoff points of PMS, TMB, and IPI were 0.33, 2.23, and 3, respectively. The survival analysis revealed that the high PMS subgroups were associated with a significantly worse PFS (*p* < 0.001) as shown in Fig. 4b. Notably, these findings were consistently found in the validation cohort (Fig. 4c, p = 0.034). The 60-month PFS rate of the high PMS subgroup in the DFCI derivation cohort (18.7%, 95% CI 10.4–33.8) and TCGA validation cohort (53.3%, 95% CI 21.4–100) were significantly worse compared to low PMS subgroups (DFCI: 95.8%, 95% CI 91.2–100; TCGA 82.6%, 95% CI 66.3–100). The progression-free Controls group had significantly lower PMS than the Cases group regardless of molecular subtypes (Supplementary Fig. S1a). Moreover, high PMS subgroups were also related to a significantly poorer PFS regardless of the molecular subtypes (Supplementary Fig. S1b–d). Specifically, the 60-month PFS rate of high PMS subgroups had a worse PFS compared to low PMS subgroups in ABC (high PMS vs low PMS: 22.2%vs 92.6%), GCB (high PMS vs low PMS: 16.7% vs 100%), and unclassified subtype (high PMS vs low PMS: 16.7%, vs 96.0%) in DFCI derivation cohort. These results indicate the potential for the proposed PMS to predict long-term prognosis across different subtypes.

**Figure 5:**
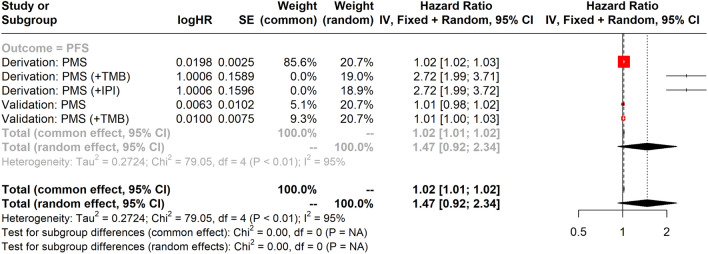
Forest plot of PMS, IPI, and TMB for PFS prognosis in both DFCI derivation and TCGA validation cohort.

Furthermore, the association between PFS and PMS, TMB, and IPI in DFCI derivation and TCGA validation cohorts was illustrated in Fig. 5 using the forest plot. The findings suggested that high PMS could be linked to a higher risk of progression in the DFCI derivation cohort (HR = 1.02, 95% CI 1.02–1.03). Similar findings were also found in the TCGA validation cohort (HR = 1.01, 95% CI 0.98–1.02), although not statistically significant. However, when TMB and IPI were added to the evaluation, the risk prediction of PMS in both cohorts was enhanced. Specifically, when TMB was involved as the covariate, PMS could obtain more significant results in PFS in both DFCI derivation (HR = 2.72, 95% CI 1.99–3.71) and TCGA validation (HR = 1.01, 95% CI 1.00–1.03) cohorts. Concordant findings were also observed for the addition of IPI in DFCI derivation (HR = 2.72, 95% CI 1.99–3.72) cohorts.

To further explore the functional role of PMS-involved genes, we first annotated the 94 selected PMS genes to the drug-gene interaction database (DGIdb). The drug-gene interaction was visualized using a chord diagram as shown in Supplementary Fig. S2. Doxorubicin was more likely associated with TP53, followed by BCL2, BRCA1, EZH2, and FCGR3A. Cyclophosphamide was more likely associated with TP53, followed by BRCA1, ITGAL, and ITGB2. In addition, prednisolone was found related to FCGR3A and ITGB1. Based on these findings, the agents in the CHOP regimen showed interaction with genes related to DNA damage, TP53, apoptosis, and immune cell functions. Further pathway analysis showed the most relevant pathways of these 94 genes were associated with DNA damage, cell cycle, inflammation, and immune cell functions (Supplementary Table S5).

## Discussion

DLBCL is characterized by heterogeneous genetic events and complicated interactions between lymphoma cells and tumor microenvironment. Through a combined approach utilizing next-generation sequencing and clinical data, we uncovered the somatic mutation profile of DLBCL with clinical significance. The most notable finding of the present study was the identification of PMS for the prediction of long-term survival. Importantly, some molecular pathways related to the constructed PMS may play an important role in the cellular functions of cancer cells including the cell cycle, DNA damage, inflammation, and immune function, were identified from the PMS.

The application of polygenic risk estimation in cancer patients has increased in popularity recently, which was driven by notable advancements in polygenic risk analysis. This approach enables the comprehensive assessment of the collective impact of multiple genetic variants associated with a specific condition^30,31^. When it comes to cancer, PMS can provide valuable insights into a patient's genetic susceptibility to disease development and progression^32^. According to the AUC evaluation, this study demonstrated that the constructed PMS involving 94 somatic mutation genes may exhibit better prediction capability than the conventional IPI score, a predictive model incorporating clinical parameters established around 30 years ago^33^. One possible explanation could be attributed to the complex genetic or epigenetic abnormalities in tumorigenesis, which can be meticulously analyzed through contemporary high-throughput sequencing technology and bioinformatics studies. Besides, previous studies suggested the accumulation of somatic mutations was related to the development of diseases, including cancer^34–36^. Hence, this analytical approach may hold the potential to provide comprehensive and invaluable prognostic information. For example, a study investigating NGS data for mutational status and its clinical relevance in patients with acute myeloid leukemia revealed a higher number of somatic mutations were associated with a worse outcome^37^. Another study utilizing NGS data to explore somatic mutation also identified certain gene mutations closely linked to overall survival in patients with anaplastic thyroid carcinoma^38^. Importantly, this study revealed the combination of genetic and clinical data can further augment the predictive capacity for long-term survival. Based on the above statement, with the advance of genetic and bioinformatic analysis, more prognostic prediction models would be developed and investigated in clinical studies^39–41^.

In the present study, we also explored the drug-gene interaction between the CHOP regimen and 94 genes in constructed PMS, the results revealed some genes like TP53, BCL2, BRCA1, EZH2, FCGR3A, ITGAL, and ITGB1 had significant interaction with therapeutic agents, which was concordant with the previous studies. For example, the existence of TP53 mutation was found to be negatively related to survival in patients with DLBCL who received R-CHOP treatment^42^. Another study indicated the expression of DNA damage response pathway and BCL-2 was linked to poorer outcome^24,43^. Moreover, the genetic and functional profile of immune cells also showed significant potential for outcome prediction^24,44^. Collectively, the constructed PMS would contain comprehensive parameters related to tumorigenesis and may provide more prognostic information.

The present study still had some limitations. First, the gene numbers in certain candidate gene sets were limited, causing potentially meaningful genes and PMS to be missed, which may have been due to a low incidence of mutation in these excluded genes. Second, the validation cohort had a comparably limited patient number and lacked consistent demographic data. In addition, approximately 30% of patients belonged to the unclassified molecular group, which might affect the result of survival analysis. Despite these issues, the identified PMS still showed satisfactory prediction performance in PFS.

Based on the above, the combination of polygenic risk estimation and clinical parameters would provide prognostic information for long-term survival in cancer patients. Regarding the perspectives, several new analytic models are also developed and investigated for mechanistic exploration and potential therapeutic therapeutic target identification. For example, a study utilizing ordinary differential equations-based modeling revealed the proteins in dynamic assembling/de-assembling of TNF signaling complexes and determination of cell death outcome^45^. Another study developed a novel mathematical model to investigate the establishment of molecular compositions within mRNA-driven protein droplets. The findings revealed that in a mixed system of two mRNAs sharing a common binding protein, the droplets preferentially assemble separately rather than colocalize, with competition occurring between them for protein recruitment^46^. Furthermore, the advances in computational biology like machine learning models can help us gain more insight into the complex crosstalk between genetic markers and related diseases^47–51^, as well as the development of genetic risk models^52,53^. Recently, the exploration of the interaction between long non-coding RNA and microRNA also provided valuable information^49,54^. Collectively, the substantial output of data produced by high-throughput sequencing represents an important breakthrough in biological research. Utilizing sophisticated bioinformatics investigative tools, the results can unveil novel mechanisms and guide subsequent functional studies.

In summary, the major contribution of this study was that we combined gene expression signatures with NGS data to identify novel molecular prognostic markers. We first identified frequent somatic mutations and then constructed the PMS, which may serve as predictors for long-term survival in DLBCL patients. The exploration of the relevant signaling pathways and genetic alterations may provide new information for further investigation to gain more insight into disease mechanisms.

### Supplementary Information

Supplementary Table S1.

Supplementary Table S2.

Supplementary Table S3.

Supplementary Table S4.

Supplementary Table S5.

Supplementary Figure S1.

Supplementary Figure S2.

## Data Availability

The data presented in this study are available downloaded from cBioPortal. 1. DFCI derivation cohort: https://cbioportal-datahub.s3.amazonaws.com/dlbcl_dfci_2018.tar.gz. 2. Validation cohort: https://cbioportal-datahub.s3.amazonaws.com/dlbc_tcga_pan_can_atlas_2018.tar.gz.
