# Supplementary material for: Prognostic mutation signature would serve as a potential prognostic predictor in patients with diffuse large B-cell lymphoma
Source: Sci Rep. 2024 Mar 14;14:6161. doi: 10.1038/s41598-024-56583-4 (PMC10940711; doi:10.1038/s41598-024-56583-4)
Supplement: Supplementary file 1 — Supplementary Table S1. [file 41598_2024_56583_MOESM1_ESM.docx]

Supplementary Table S1. Somatic mutation of specific genes according to molecular subtype.

| Gene | ABC, n= 45 | GCB, n= 37 | Not classified, n= 38 | *P* |
| --- | --- | --- | --- | --- |
| Somatic mutation |  |  |  |  |
| BCL2 | 0 (0.0%) | 11 (29.7%) | 18 (47.4%) | <0.001 |
| TP53 | 10 (22.2%) | 10 (27.0%) | 7 (18.4%) | 0.671 |
| MYC | 1 (2.2%) | 2 (5.4%) | 4 (10.5%) | 0.294 |
| MCM5 | 3 (6.7%) | 0 (0.0%) | 1 (2.6%) | 0.384 |
| TSHZ3 | 3 (6.7%) | 0 (0.0%) | 3 (7.9%) | 0.282 |
| KLHL6 | 3 (6.7%) | 3 (8.1%) | 5 (13.2%) | 0.626 |
| MYD88 | 16 (35.6%) | 5 (13.5%) | 1 (2.6%) | <0.001 |
| CD79B | 11 (24.4%) | 5 (13.5%) | 3 (7.9%) | 0.108 |
| CREBBP | 4 (8.9%) | 8 (21.6%) | 13 (34.2%) | 0.018 |

ABC, activated B-cell type. GCB, germinal center B-cell type.
