# Supplementary material for: Prognostic mutation signature would serve as a potential prognostic predictor in patients with diffuse large B-cell lymphoma
Source: Sci Rep. 2024 Mar 14;14:6161. doi: 10.1038/s41598-024-56583-4 (PMC10940711; doi:10.1038/s41598-024-56583-4)
Supplement: Supplementary file 2 — Supplementary Table S2. [file 41598_2024_56583_MOESM2_ESM.docx]

Supplementary Table S2. The clinicopathological characteristics, TMB, and survival status of the derivation cohort (n=120).

| Characteristics | Overall,  n= 120 | Controls^1^,  n= 68 | Cases^2^,  n= 52 | *P* |
| --- | --- | --- | --- | --- |
| Age at diagnosis (years) | 66 (26-88) | 66 (26-82) | 69 (30-88) | 0.051 |
| Gender |  |  |  | 0.056 |
| Female | 55 (45.8%) | 26 (38.2%) | 29 (55.8%) |  |
| Male | 65 (54.2%) | 42 (61.8%) | 23 (44.2%) |  |
| Molecular subtype |  |  |  | 0.423 |
| ABC | 45 (37.5%) | 25 (36.8%) | 20 (38.5%) |  |
| GCB | 38 (31.7%) | 19 (27.9%) | 19 (36.5%) |  |
| Unclassified | 37 (30.8%) | 24 (35.3%) | 13 (25.0%) |  |
| IPI score |  |  |  | 0.125 |
| Low (0-1) | 47 (39.2%) | 31 (45.6%) | 16 (30.8%) |  |
| Intermediate (2-3) | 57 (47.5%) | 31 (45.6%) | 26 (50.0%) |  |
| High (4-5) | 16 (13.3%) | 6 (8.8%) | 10 (19.2%) |  |
| TMB (mut/MB) | 2.4 (0.1-9.0) | 2.6 (0.1-9.0) | 2.2 (0.6-6.7) | 0.505 |
| Somatic mutations |  |  |  |  |
| BCL2 | 29 (24.2%) | 15 (22.1%) | 14 (26.9%) | 0.537 |
| TP53 | 27 (22.5%) | 12 (17.6%) | 15 (28.8%) | 0.145 |
| MYC | 7 (5.8%) | 3 (4.4%) | 4 (7.7%) | 0.465 |
| MCM5 | 4 (3.3%) | 4 (5.9%) | 0 (0.0%) | 0.132 |
| TSHZ3 | 6 (5.0%) | 2 (2.9%) | 4 (7.7%) | 0.401 |
| KLHL6 | 11 (9.2%) | 4 (5.9%) | 7 (13.5%) | 0.205 |
| MYD88 | 22 (18.3%) | 13 (19.1%) | 9 (17.3%) | 0.800 |
| CD79B | 19 (15.8%) | 10 (14.7%) | 9 (17.3%) | 0.699 |
| CREBBP | 25 (20.8%) | 14 (20.6%) | 11 (21.2%) | 0.940 |
| All-cause mortality | - | - | 42 (80.8%) | <0.001 |

The *P value* was estimated using the Wilcoxon rank-sum test, chi-squared test, or Fisher’s exact test.

^1^ Controls: patients without progression, relapse or died within follow-up duration.

^2^ Cases: patients with relapse/progression of lymphoma or died within follow-up duration.
