# Supplementary material for: Prognostic mutation signature would serve as a potential prognostic predictor in patients with diffuse large B-cell lymphoma
Source: Sci Rep. 2024 Mar 14;14:6161. doi: 10.1038/s41598-024-56583-4 (PMC10940711; doi:10.1038/s41598-024-56583-4)
Supplement: Supplementary file 3 — Supplementary Table S3. [file 41598_2024_56583_MOESM3_ESM.docx]

Supplementary Table S3. The clinicopathological characteristics, TMB, and survival status of the TCGA validation cohort (n= 25).

| Characteristics | Overall,  n=25 | Controls^1^,  n=18 | Cases^2^,  n=7 | *P* |
| --- | --- | --- | --- | --- |
| Age at diagnosis (years) | 54.0 (45.0, 67.0) | 53.0 (45.3, 66.0) | 64.0 (50.5, 66.5) | 0.486 |
| Gender |  |  |  | 0.355 |
| Female | 16 (64.0%) | 10 (55.6%) | 6 (85.7%) |  |
| Male | 9 (36.0%) | 8 (44.4%) | 1 (14.3%) |  |
| Molecular subtype |  |  |  | 0.534 |
| Mediastinal (Thymic) | 3 (12.0%) | 3 (16.7%) | 0 (0.0%) |  |
| DCBCL (NOS) | 22 (88.0%) | 15 (83.3%) | 7 (100.0%) |  |
| TMB (mut/MB) | 2.5 (1.1, 5.1) | 2.8 (1.7, 5.5) | 1.9 (0.5, 3.1) | 0.300 |
| All-cause mortality | 3 (12.0%) | 1 (5.6%) | 2 (28.6%) | 0.180 |

The *P value* was estimated using the Wilcoxon rank-sum test, chi-squared test, or Fisher’s exact test.

^1^ Controls: patients without progression, relapse or died within follow-up duration.

^2^ Cases: patients with relapse/progression of lymphoma or died within follow-up duration.
