# Supplementary material for: Prognostic mutation signature would serve as a potential prognostic predictor in patients with diffuse large B-cell lymphoma
Source: Sci Rep. 2024 Mar 14;14:6161. doi: 10.1038/s41598-024-56583-4 (PMC10940711; doi:10.1038/s41598-024-56583-4)
Supplement: Supplementary file 6 — Supplementary Figure S1. [file 41598_2024_56583_MOESM6_ESM.pdf]

Supplementary Figure S1. (a) Boxplot of PMS across different subtypes in DFCI derivation cohort. Kaplan–Meier plot of low-and high-PMS in DFCI derivation cohort according to (b) ABC, (c) GCB, and (d) unclassified subtypes.

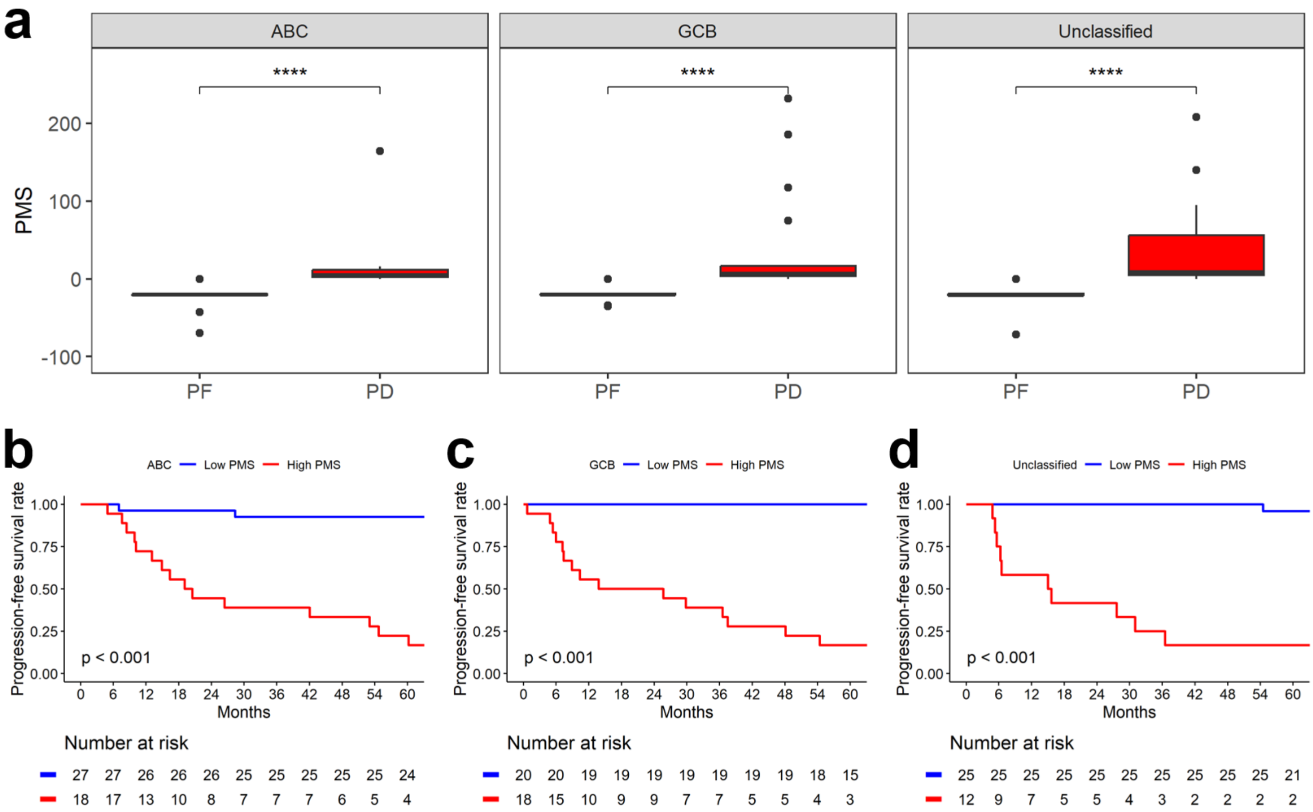

ABC (n=45)
Derivation cohort
High PMS (n=18)
12-month = 72.2% (95%CI=54.2-96.2)
24-month = 44.4% (95%CI=26.5-74.5)
36-month = 38.9% (95%CI=24.2-51.9)
48-month = 33.3% (95%CI=17.0-64.1)
60-month = 22.2% (95%CI=9.4-52.7)

Low PMS (n=27)
12-month = 96.3% (95%CI=89.4-100)
24-month = 96.3% (95%CI=89.4-100)
36-month = 92.6% (95%CI=83.2-100)
48-month = 92.6% (95%CI=83.2-100)
60-month = 92.6% (95%CI=83.2-100)

GCB (n=38)
Derivation cohort
High PMS (n=18)
12-month = 55.6% (95%CI=36.6-84.0)
24-month = 50.0% (95%CI=31.5-79.4)
36-month = 38.9% (95%CI=21.8-69.4)
48-month = 27.8% (95%CI=13.2-58.5)
60-month = 16.7% (95%CI=5.9-46.8)

Low PMS (n=20)
12-month = 100% (95%CI=100-100)
24-month = 100% (95%CI=100-100)
36-month = 100% (95%CI=100-100)
48-month = 100% (95%CI=100-100)
60-month = 100% (95%CI=100-100)

Unclassified (n=37)
Derivation cohort
High PMS (n=12)
12-month = 58.3% (95%CI=36.2-94.1)
24-month = 41.7% (95%CI=21.3-81.4)
36-month = 25.0% (95%CI=9.4-66.6)
48-month = 16.7% (95%CI=4.7-5.9)
60-month = 16.7% (95%CI=4.7-5.9)

Low PMS (n=25)
12-month = 100% (95%CI=100-100)
24-month = 100% (95%CI=100-100)
36-month = 100% (95%CI=100-100)
48-month = 100% (95%CI=100-100)
60-month = 96.0% (95%CI=88.6-100)
