# Supplementary material for: Prognostic mutation signature would serve as a potential prognostic predictor in patients with diffuse large B-cell lymphoma
Source: Sci Rep. 2024 Mar 14;14:6161. doi: 10.1038/s41598-024-56583-4 (PMC10940711; doi:10.1038/s41598-024-56583-4)
Supplement: Supplementary file 7 — Supplementary Figure S2. [file 41598_2024_56583_MOESM7_ESM.pdf]

Supplementary Figure S2. Drug-gene interaction chord diagram for PMS genes and CHOP-like regimen.

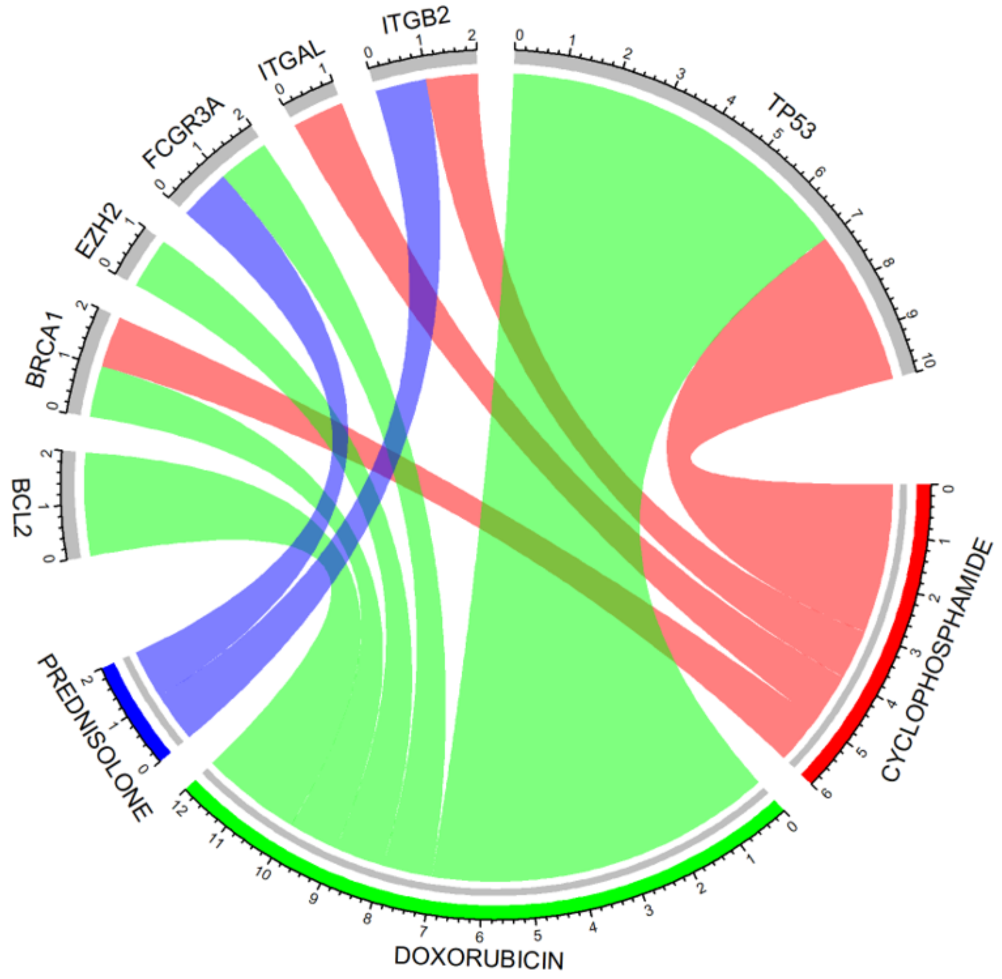

Drug	Gene	PMIDs
CYCLOPHOSPHAMIDE	BRCA1	25589624
CYCLOPHOSPHAMIDE	ITGB2	9523002
CYCLOPHOSPHAMIDE	TP53	17388661
CYCLOPHOSPHAMIDE	TP53	16243804
CYCLOPHOSPHAMIDE	TP53	26438783
CYCLOPHOSPHAMIDE	ITGAL	9389690
DOXORUBICIN	BRCA1	12698198
DOXORUBICIN	TP53	25658463
DOXORUBICIN	TP53	21399868
DOXORUBICIN	TP53	16243804
DOXORUBICIN	TP53	23165797
DOXORUBICIN	TP53	26826118
DOXORUBICIN	TP53	22698404
DOXORUBICIN	TP53	9569050
DOXORUBICIN	BCL2	10914739
DOXORUBICIN	FCGR3A	1830717
DOXORUBICIN	BCL2	16749867
DOXORUBICIN	EZH2	25605023
PREDNISOLONE	ITGB2	8976974
PREDNISOLONE	FCGR3A	17329922
